# Modular training resources for bioimage analysis

**DOI:** 10.1111/jmi.70133

**Published:** 2026-06-18

**Authors:** Christian Tischer, Antonio Zaccaria Politi, Tim‐Oliver Buchholz, Elnaz Fazeli, Nicola Gritti, Aliaksandr Halavatyi, Sebastián González‐Tirado, Julian Hennies, Toby Hodges, Arif Khan, Dominik Kutra, Stefania Marcotti, Bugra Oezdemir, Felix Schneider, Martin Schorb, Anniek Stokkermans, Yi Sun, Nima Vakili

**Affiliations:** ^1^ Data Science Centre European Molecular Biology Laboratory Heidelberg Germany; ^2^ Facility for Light Microscopy Max Planck Institute of Multidisciplinary Sciences Göttingen Germany; ^3^ Facility for Advanced Imaging and Microscopy Friedrich Miescher Institute for Biomedical Research Basel Switzerland; ^4^ Biomedicum Imaging Unit, Faculty of Medicine and HiLIFE University of Helsinki Helsinki Finland; ^5^ Mesoscopic Imaging Facility European Molecular Biology Laboratory Barcelona Spain; ^6^ Advanced Light Microscopy Facility European Molecular Biology Laboratory Heidelberg Germany; ^7^ Institute for Computational Biomedicine, Heidelberg University Heidelberg Germany; ^8^ EMBL Imaging Centre European Molecular Biology Laboratory Heidelberg Germany; ^9^ The Carpentries, c/o Community Initiatives Oakland California USA; ^10^ Cell Biology and Biophysics European Molecular Biology Laboratory Heidelberg Germany; ^11^ Randall Centre for Cell and Molecular Biophysics, King's College London London UK; ^12^ Euro‐BioImaging Bio‐Hub, EMBL Heidelberg Germany; ^13^ Genome Biology European Molecular Biology Laboratory Heidelberg Germany; ^14^ Electron Microscopy Core Facility European Molecular Biology Laboratory Heidelberg Germany; ^15^ Hubrecht Institute Utrecht the Netherlands

**Keywords:** image analysis, microscopy, open‐access, teaching

## Abstract

Modern microscopy enables us to measure structural and dynamical properties of many biological processes and is therefore an indispensable research tool. However, the amount and complexity of the produced imaging data is steadily increasing. Thus, handling the data as well as reproducibly and automatically extracting accurate scientific information require dedicated ‘bioimage analysis’ expertise. To facilitate the dissemination of this ubiquitously required expertise we developed an open‐access bioimage analysis training resource. The resource is designed to help trainers to design and run courses on bioimage analysis for life scientists. The material is modular where each module covers one concise topic and provides corresponding activities using microscopy images from biological samples. The activities can be executed using various popular software packages (e.g. ImageJ, Python). The material is hosted on a public software repository allowing the bioimaging community to readily contribute new training modules or improve existing modules. Within the last 3 years, the material has been used by several trainers in numerous courses and continuously improved.

## 1 INTRODUCTION

Microscopy is a key technology driving biological research and a large and diverse software ecosystem exists to analyse microscopy images. Software solutions can be open‐source or commercial, and vary from general to specific use‐cases, ease of use, levels of documentation, and accessibility.^1^ Despite these available tools, the complexity and size of the corresponding imaging data can make it challenging to efficiently extract bio‐physically meaningful information from the acquired images in a quantitative and reliable/reproducible manner. As a consequence, the bio‐computational discipline ‘bioimage analysis’ has emerged.^2^, ^3^ Scientists performing bioimage analysis have a diverse background and need to acquire a theoretical foundation on image formation as well as image processing and analysis algorithms. Furthermore, scientists must acquire the necessary skills to effectively and adequately use various software tools for image analysis tasks.

During the last decade, online resources for self‐study have emerged in the form of videos, slides, interactive books, and Massive Open Online Courses (MOOCs).^4^, ^5^, ^6^, ^7^, ^8^ In addition, numerous in‐person or online bioimage analysis courses have been delivered and sometimes recorded.^9^, ^10^ Live online or in‐person courses are very valuable as they provide a platform for networking, interacting with expert trainers, and addressing specific questions with respect to learners’ needs. We found that an open‐access resource allowing bioimage analysis trainers to efficiently design and deliver such courses to various target audiences would be helpful and is currently missing.^11^, ^12^ Such a resource should help and simplify the much‐needed dissemination of bioimage analysis skills.

To address those needs we started in early 2019 to create an open‐access online resource for bioimage analysis training accessible via https://neubias.github.io/training‐resources/.^13^ From 2021 to 2024, this resource has been used in at least 25 courses with in total nearly 600 participants (Table S1). With this publication we aim to further spread the use of this material and also kindly invite the bioimaging and microscopy community to contribute to any aspect of this resource.

In the following sections we will describe the rationale, content, implementation details and usage instructions.

## 2 TRAINING RESOURCE DESIGN AND ORGANISATION

We aimed for a resource to facilitate delivering in‐person and online live courses. The material should include activities to illustrate use cases. Given the large number of image analysis software, we wanted to separate an activity from a particular software implementation. Finally, the material should allow bioimage analysts to tailor courses for different target audiences. This led to the following design constraints: open‐access online hosting of the material, modularity, interactivity, usability within a live and online context, and easy extension by the community.

### 2.1 Design principles

We found that existing online material is primarily conceived as self‐study resources and does not yet fulfil all our prerequisites. While a simple collection of slides could be used within a live teaching context, they are rather static, often lack integrated activities, and are hard to develop/maintain as a community. Pre‐recorded videos are inappropriate for a live, interactive setting and also lack integrated activities. MOOCs provide comprehensive material often enriched with videos, exercises, and assessments that are best suited for self‐paced study.^7^, ^8^ The interactive book by P. Bankhead^4^ contains comprehensive explanations, example images, and activities, representing a good reference for bioimage analysis concepts. While all the aforementioned materials are great resources, they impose a pre‐defined order of topics and lack modularity. Modifications or additions of new concepts may therefore require major revisions; thus, in our view, the existing resources are not designed to support agile contributions by a community of bioimage analysts.

We decided to base our training resource on the didactically excellent training material of *The Carpentries* (https://carpentries.org/).^14^, ^15^ A Carpentries expert has been a critical contributor to the initial development of the bioimage analysis training resource. Among other things, lessons by *The Carpentries* are aimed at novices as well as experts and gradually build knowledge, avoiding too much information at once. A lesson is composed of several so‐called episodes, lasting for 20 to 60 min with clearly stated learning objectives. Each episode includes activities suited for live demonstration/live‐coding, so to explain every step of the process, and formative assessments to identify misconceptions. *The Carpentries* provide a course on image‐processing with python that can be used in a live context with a variety of example images and activities.^16^

Our material differs from *The Carpentries*' primarily in its increased modularity and specific application to bioimaging data. Unlike *The Carpentries*, which delivers predefined lessons, we designed small, independent training modules (reminiscent to *Carpentries* episodes) that can be flexibly assembled into various courses—differing in length, target audience, and software packages covered (Figure 1). To manage and optimise cognitive load,^17^ each training ‘module’ focuses on just one or very few new concepts that should be taught within 30–45 min. This inherently restricts the complexity or number of concepts included within a single module. A ‘concept’ in this context refers to a generic, commonly used principle or method in bioimage analysis that is agnostic to specific software implementations, such as ‘object shape measurement’. A module is designed to provide all the necessary information to effectively motivate, explain (e.g., through figures, concept maps), and apply, in so called activities (e.g., using example images and code), its content. In the activities of each module, we address problems specific to bioimaging by using microscopy images of biological samples.

**FIGURE 1:**
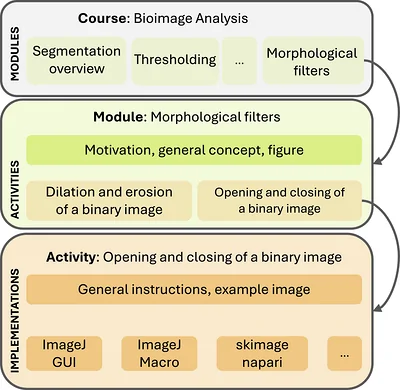
Training resource design. Hierarchy of the bioimage analysis training course. A course is designed by assembling several existing modules. Each module explains a concept/method and provides activities. An activity has general instructions, an example image, and different software implementations.

### 2.2 Module structure

The above design principles, refined via repeated teaching of different modules, led to the subdivision of each module into nine distinct sections, as detailed in Figure 2 and Table 1. The *Prerequisites* section indicates which modules should be learned first. The other four sections serve to teach the concept in a lecture style presentation, where the *Concept Map* and a *Figure* are used as a visual support for the instructor to explain the concept to the learners. Some additional detailed explanations could be added after the main figure. The main section of a module is *Activities* that demonstrates different applications of the concepts that are taught in the module. Each activity is described in general software‐agnostic terms and has linked example data, typically one or several microscopy images of biological samples, that can be readily used (url or drag‐and‐drop into the software). The instructor and learner can then select instructions for specific software packages (Figure 2D and E). This separation of general concepts from specific implementations was a major design decision for this resource. This makes it possible to continuously add new software implementations of an activity, without needing to change much of the generic teaching content. In addition, this design also increases the didactic quality of the material, because it forces the trainer and students to disentangle universally applicable concepts and methods such as ‘connected component analysis’ and ‘object shape measurements’, from specific implementations such as ImageJ's Particle Analyzer^18^ or scikit‐image's regionprops^19^.

**FIGURE 2:**
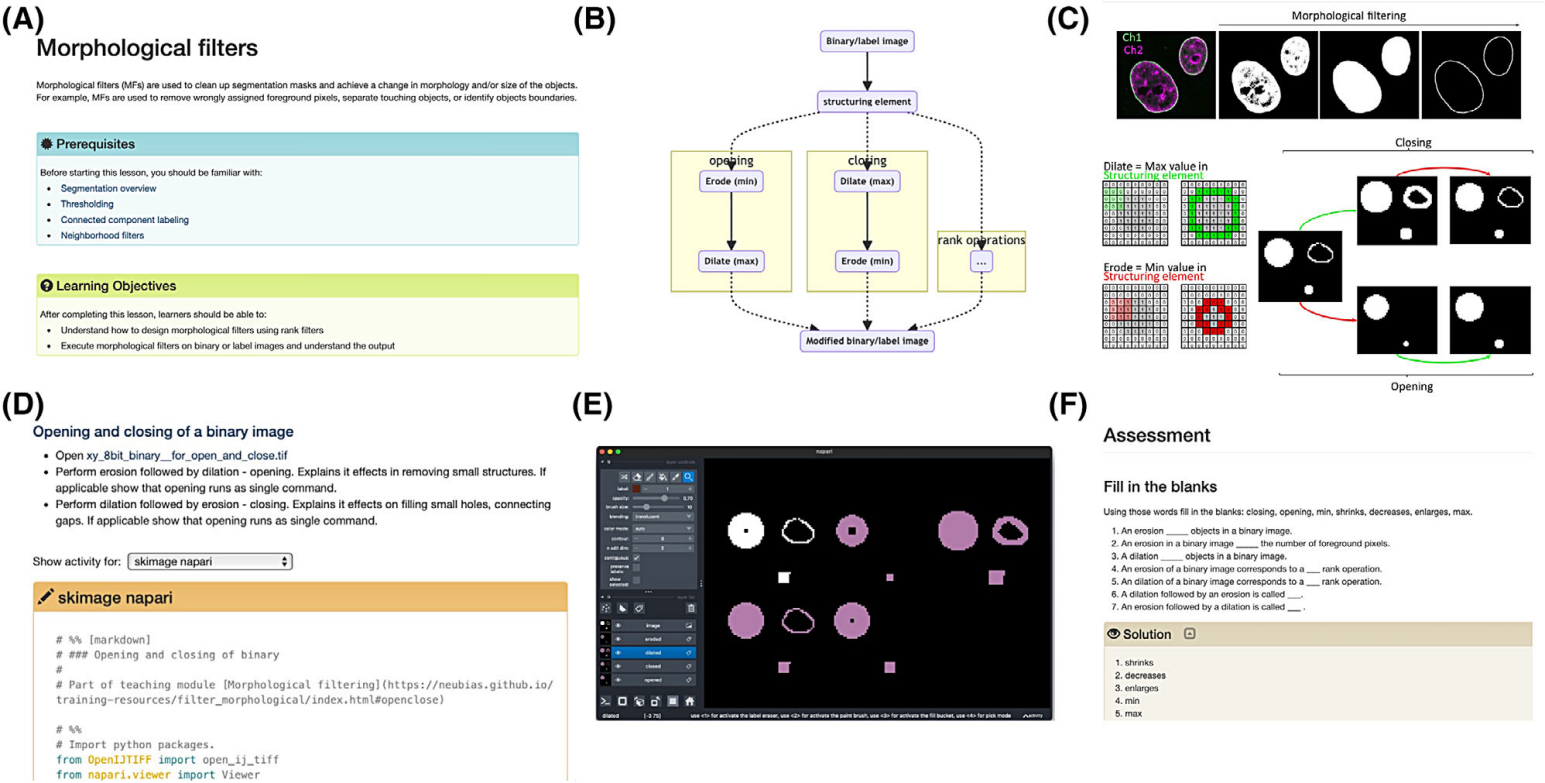
Structure of a training module. This figure shows screenshots of a module website and activity. (A) A module starts with a motivation paragraph, providing the why and what will be learned during the module. Knowledge required to follow a given module is provided via links to other modules (*Prerequisites*). *Learning objectives* summarises the goal of the module. (B) *Concept Map* and (C) *Figure* visuals provide an overview of the key concepts. They can be used to support the lecturing part of a module. Additional explanations may appear after the main figure (not shown). In *Activities* (D) the activity instruction and its implementations provide the necessary information to showcase a concept using a specific software (e.g. python and scikit‐image/napari). (E) Example output generated when executing an activity. (F) Formative *Assessments* are provided at the end of a module to reinforce what has been learned. Finally, a *Follow‐up material* section with links to other modules or external material closes the module (not shown).

**TABLE 1:** Training module sections.

Module section	Explanation
Motivation	Explains why it is important to acquire these skills
Prerequisites	Contains links to other modules of our material, defining the content that learners should know before starting
Learning objectives	Defines the skills that learners will acquire
Concept map	A schematic drawing that defines the core new concepts that are taught
Figure	A figure illustrating the teaching content
Explanations (optional)	Additional details typically used to help self‐study and add mathematical context
Activities	Interactive instructions for applying the concepts taught in various software platforms
Assessment	Questions and answers for testing the conceptual understanding
Follow‐up material	Links to suitable follow‐up training modules and extra‐curricular material

*Note*: A training module is composed of different sections to introduce the concept, provide activities to illustrate the concept, and further reading.

The subsequent *Assessment* section (Figure 2F) comprises short quizzes that allow the participants to test their understanding of the main concepts. For instance, a question and answer could be: ‘Q: What is the typical data type of a label mask image? A: Typically, label mask images are of positive integer data types (unsigned integer 8 or 16 bit) with zero being the background label’. Finally, the *Follow‐up Material* section provides links to additional modules that can be learned after the current module or external resources.

In our experience, this predefined module structure significantly facilitates the addition of new material. While it still forces the contributor to carefully think about what they actually want to teach in the module, it frees one from thinking too much about how to structure and format the material.

### 2.3 Resource content

Over the last 6 years we created ∼62 teaching modules (Table 2, Table S2). Videos S1–S3 illustrate the content of the training material and how to teach a module. Typically, a specific set of modules is taught within a course. The online resource provides 9 example courses (Table S2) of variable duration.

**TABLE 2:** List of modules.

Module title	Learning objectives
Automatic thresholding	Understand how an image histogram can be used to derive a threshold. Apply automatic threshold to distinguish foreground and background pixels.
Batch exploration of segmentation results	Use various tools to efficiently inspect segmented images and corresponding object measurements.
Big image data formats	Understand the concepts of lazy‐loading, chunking and scale pyramids. Understand some file formats that implement chunking and scale pyramids.
Cloud based batch analysis	Understand how to leverage cloud computing platforms, such as Galaxy, for efficient bioimage batch analysis.
Cloud based interactive analysis	Understand interactive analysis platforms, including virtual desktops, Galaxy interactive tools, and Jupyter notebooks for bioimage analysis. Run bioimage tools, such as CellPose on virtual desktops. Run Galaxy Interactive Tools for bioimage analysis.
Connected component labelling	Understand how objects in images are represented as a label mask image. Apply connected component labelling to a binary image to create a label mask image.
Convolutional filters	Understand the basic principle of a convolutional filter. Apply convolutional filters to an image.
Correlative image rendering	Understand how heterogeneous image data can be mapped from voxel space into a global coordinate system. Understand how an image viewer can render a plane from a global coordinate system.
Data types	Understand that images have a data type which limits the values that the pixels in the image can have. Understand common data types such as 8‐bit, 12‐bit and 16‐bit unsigned integer.
Digital image basics	Understand that a digital image is represented as an N‐dimensional array. Visualise an image and quantitatively inspect the image (array) elements (pixels, voxels).
Display settings	Understand how the numerical pixel values of an image are transformed into bright and colourful images that you see on display screen. Understand what a colourmap is and why adjusting it is useful. Understand how perception about image content changes on viewing it by altering contrast and colourmaps. Use display settings responsibly for scientific purposes.
Distance transform	Understand how to use distance transform to quantify morphology of objects. Understand how to use distance transform to quantify distance between objects. Understand approximate nature of distance transform.
Flat‐field correction	Formulate the mathematical relationship between the raw image and the corrected image. Evaluate methods to obtain a flat‐field reference (calibration slide or retrospective computation). Perform a flat‐field correction and evaluate the results.
Global background correction	Measure the background in an image. Apply image math to subtract a background intensity value from all pixels and understand that the output image should have a floating point data type.
Illumination and shading artefacts	Identify global shading patterns caused by, e.g. Gaussian illumination. Understand implication for intensity measurements.
Image data formats	Open various image files formats. Understand the difference between image data and metadata.
Image sampling	Explain how spatial and axial sampling discretise a continuous optical image. Identify under‐sampling artefacts such as aliasing and loss of structural information. Recognise over‐sampling and its practical costs (data volume, phototoxicity, acquisition time). Understand that the biological question guides the required sampling.
Imaging thick biological samples	Understand that the geometry and optical properties of your biological specimen can have a large influence on the measured intensities.
Local background correction	Understand how to use image filters for creating a local background image. Use the generated local background image to compute a foreground image.
Median filter	Understand in detail what happens when applying a median filter to an image.
Morphological filters	Understand how to design morphological filters using rank filters. Execute morphological filters on binary or label images and understand the output.
Multichannel images	Understand/visualise different image channels.
N‐dimensional images	Explore and view the different dimensions image data can have.
Neighbourhood filters	Understand the basic principle of a neighbourhood filter. Apply basic neighbourhood filters to an image.
Object filtering	Remove objects from a label mask image.
Object intensity measurements	Understand the correct biophysical interpretation of the most common object intensity measurements. Perform object intensity measurements, including background subtraction.
Object shape measurements	Understand shape measurements and their limitations. Perform shape measurements on objects.
OME‐TIFF	Understand the basic concept of the TIFF image data format. Understand that there are various TIFF variants, such as ImageJ‐TIFF and OME‐TIFF. Create OME‐TIFF files. Convert other image data into OME‐TIFF files. Inspect OME‐TIFF image data and metadata.
OME‐Zarr	Understand the OME‐Zarr image file format. Render cloud (S3 object store) hosted OME‐Zarr image data. Access the pixel values of cloud hosted OME‐Zarr image data. Apply basic image processing on cloud hosted OME‐Zarr image data.
Optical sectioning	Understand the effect of optical sectioning in an image.
Optimal sampling	Be able to compute the optimal pixel size according to Nyquist given a resolution limit. Decide which pixel size is required to visualise a structure of a specific size.
Point spread function	Identify PSF‐induced artefacts in microscopy images, specifically axial elongation (Z‐distortion) and lateral blurring. Predict the impact of the PSF on quantitative measurements, including how it inflates object size and dilutes signal intensity. Understand how microscope hardware configurations change the PSF.
Projections	Project multi‐dimensional image data into lower dimensions. Understand the differences between projection modes such as max, sum, and mean.
Resolution	Understand how resolution is affected by the size of the PSF. Understand how physical parameters of the microscope affect the resolution.
Sample preparation artefacts	Understand that sample preparation will affect the final imaging result. Understand which type of artefacts come from sample preparation.
Segmentation inspection	Visually inspect segmentation quality by overlaying segmentations on the raw image. Compute IoU for semantic segmentation by converting label masks to binary masks. Compare instance segmentations using object counts and mean object area.
Segmentation overview	Understand the difference between instance and semantic segmentation.
Skeletonisation	Apply a skeletonisation algorithm to a binary image to view its internal skeleton. Count the number of branches and branch lengths to obtain morphological information from the image.
Smart microscopy targeted imaging	Understand basic feedback microscopy workflow for automatically finding target objects and re‐imaging them at high resolution. Understand essential steps in constructing and testing such a workflow. Learn which use cases would be appropriate for such workflow and possible limitation.
Spatial calibration	Understand that a pixel index is related to a physical coordinate. Understand that a spatial calibration allows for physical size measurements.
Spherical aberrations	Intensity Loss (Dimming): Understand why light fails to converge at a single focal point, leading to a dimmer and less contrasting signal. Learn how the difference between your immersion oil and your sample environment triggers these aberrations. Z‐Shift and Scaling: Identify why objects appear at the wrong depth or seem elongated/shortened in 3D space.
Statistical (rank) filters	Understand conceptually how local statistical (rank) filter work. Apply statistical filters to images.
Stitching of tiled images	Learn how to stitch tiled images into a single large image. Learn which parameters affect the quality of the results.
Thresholding	Describe the relationship between an intensity image and a derived binary image. Apply threshold to segment an image into foreground and background regions.
Tool installation	Install the software that is required to execute the activities in this training material.
Volume rendering	Understand the concepts and some methods of 3‐D rendering. Appreciate that 3‐D rendering can be challenging for some data. Perform basic volume rendering using a software tool.
Volume slicing	Create slice views of volumetric image data. Master different ways of dealing with anisotropic voxels.
Watershed	Understand the concept of watersheds in image analysis. Understand that a watershed algorithm is often applied to a distance map to split objects by their shape. Be able to run a watershed algorithm in an image analysis platform.
Noisy object segmentation and filtering in 2D	Create an image analysis workflow comprising image denoising and object filtering.
Nuclei and cells segmentation	Segment cells and nuclei, using nuclei as seeds for watershed segmentation of the cells.
Nuclei segmentation and shape measurement	Create a basic image analysis workflow. Understand that bioimage analysis workflows consist of a sequence of image analysis components. Segment nuclei in a 2D image and measure their shapes and understand the components (concepts and methods) that are needed to accomplish this task. Draw a biophysically meaningful conclusion from applying an image analysis workflow to a set of images.
Quantitative image inspection and presentation	Quantitatively inspect and present fluorescent microscopy images.
Batch processing	Automatically process a number of images.
Coding with LLMs	Use a large language model (LLM) to create bioimage analysis code. Use a LLM to understand bioimage analysis code.
Functions	Wrap a piece of code into a function. Understand the skeleton of a function.
Handling input parameters	Organise script parameters in the code such that can be easily adapted. Create dialog boxes for fetching script parameters.
Loops	Use for loops to repeat operations multiple times. Running a script for multiple files.
Output saving	Save measurements as a table. Save ROIs. Save output label mask.
Recording a script	Record graphical user interface (GUI) actions into a script.
Running a script	Understand that a script is a single text file that is written in a specific scripting language. Understand the basic building blocks of a script, i.e. what happens in each line. Run a bioimage analysis script. Modify a bioimage analysis script.
Strings and paths	Construct complex strings, e.g. to produce log messages and create file paths. Automatically create paths for saving the results of the analysis of an input image.
Variables	Understand difference between variable name, value, type, and storage. How to use variables in functions.

*Note*: List of consolidated modules in the resource (as of May 2026).

The current material covers content typically taught in introductory and intermediate bioimage analysis courses. Among others, the resource includes modules on image data handling and the understanding of the different data formats typical for microscopy, image data inspection, image filtering and thresholding, segmentation and measurements, as well as basic scripting, batch processing, and cloud‐based computing. Understanding how an image formed is also important for the analysis and therefore we included several modules on microscopy image formation and possible related artefacts (e.g. uneven illumination, spherical aberrations). We also included so‐called workflows, where learners, starting from example images, apply a sequence of methods to solve an image analysis task and obtain a result table. Finding suitable example image data for teaching a certain concept is a major effort. The repository contains small example image data, typically microscopy data of biological samples, that have been carefully selected to fulfil this need. The activities are designed for different levels of understanding and complexity.

## 3 HOW TO USE THE TRAINING RESOURCE

The use of the resource is graphically shown in Figure 3. The resource is designed to deliver courses in an online, in‐person or hybrid setup. The resource is accessible from any web‐browser via the site https://neubias.github.io/training‐resources/ (Figure 3A). The teacher can design a course using the module graph (Figures 3B and S1). Using this graph one can navigate to the respective module or highlight dependencies. Seeing those dependencies facilitates bioimage analysts putting together a consistent course. For instance, if the goal of the course is for learners to be able to perform a workflow to segment and quantify a noisy image (Figure S1), then a course should include convolutional filtering, object shape measurements, connected component analysis and other modules highlighted in green in (Figure S1). Alternatively, the home page provides a set of courses, with their respective duration, number of modules, and number of European Credit Transfer System (ECTS) credits. The courses have variable duration and range from understanding images and different image formats, up to bioimage analysis, batch processing, cloud computing, and microscopy image formation (see Table S2). Clicking on a course opens a page with the modules to be taught within the course in their correct order. All modules can also be accessed in an alphabetical order; a *Tool installation* module explains how to install the relevant software.

**FIGURE 3:**
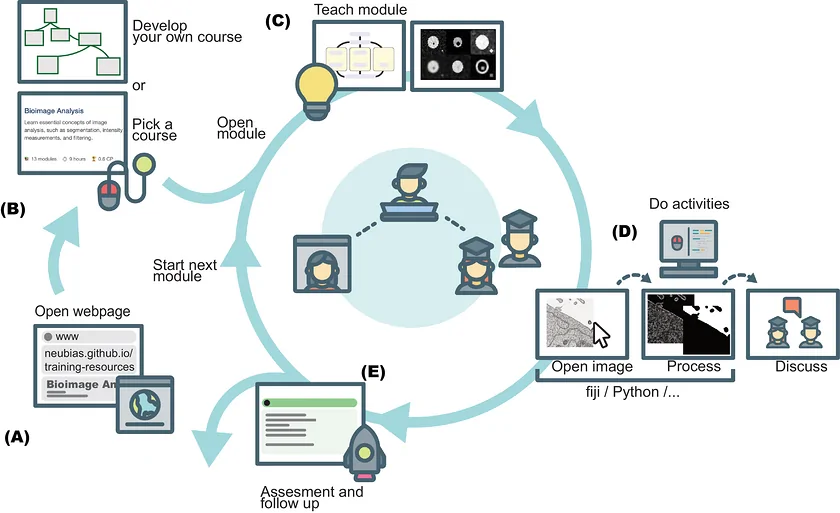
How to use the resource. Use the training resource to teach in person and/or online. (A) Teacher and learners access the course material via the website. (B) One can assemble a new course using the dependency network or use pre‐existing courses. The modules in a course are taught one after the other. (C) Each module starts by explaining the concept using the provided concept map and figure. (D) The teacher and learners execute one (or more) activities using one of the example images, typically a microscopy image of a biological sample, and discuss the results. (E) Additional activities and follow‐up material allow to further reinforce the learned concepts.

The training resource has been used to conduct numerous courses (online and in person).The training material has also been used to support scientific project discussions within an imaging facility context. By going through a set of specific modules relevant to the project, the bioimage analysis expert can help a facility user to clarify the questions, identify possible solutions, and eventually enable the facility user to solve the problem on their own.

## 4 HOW TO RUN A COURSE

Essentially, a course is a set of modules taught one after the other (Figure 3C and D). In addition, it is recommended to plan an upfront trouble‐shoot session to solve tool installation issues and a get‐together session, where participants introduce themselves and describe their image analysis needs and motivation to join the course. At the end of the course, to further reinforce what has been learned, course organisers can plan an extra ½‐ to 1‐day project time. During this time, learners apply what they learned in groups on selected projects.

In our hands, courses lasted between 0.5 and 3 days and included 3 to 18 teaching modules. Teaching one module took around 45 min such that, including breaks, about 6 modules could be taught in 1 day. The choice of modules depends on the audience, the goal of the course, and the available time. To tailor the course to the audience's needs one can ask participants to fill in a questionnaire at registration and/or ask to submit project slides. After identifying the course goal and level of the audience an instructor can use one of the consolidated courses as given on the home page of the training material (Figure 3B) or design one's own course. In the latter case, the dependency network (Figures 3B and S1) helps to identify a consistent sequence of modules, which can be shared with the learners as a document containing the links of the selected modules.

## 5 HOW TO TEACH A MODULE

To teach a module both the instructor and the learners open the corresponding website. It can be useful to have two computer screens, one with the teaching material website and another with the software that is used to conduct the activities. Each module starts with a lecturing part, where the module web page is screen shared, followed by interactive activities where the software screen is shared. Using the sections on *Motivation* and *Learning Objectives* the instructor engages the audience by explaining in which context the module content is useful and which practical skills are going to be taught. The lecture continues, by explaining the main concepts, for example, using a drawing board, discussing the module figure and/or the concept map (Figures 2A–C and 3C).

Next, the conceptual knowledge is applied within one or more *Activities* (Figures 2D and E and 3D). An activity typically starts with opening a microscopy image of a biological sample and performing some operations illustrating the concept. During the activity part, the instructor shares the screen containing the respective software tool. The module web page is best kept on a separate screen and is used to guide the instructor throughout the activity. Images can be drag and dropped or downloaded from the module web site to the respective software. The pace of the activity should be slow enough so that learners can click‐along (for a GUI based software) or type‐along (for coding). This allows the instructor to provide additional explanations on the different commands and learners to slowly deepen their understanding on a practical level.^15^ Finally, learners could test their conceptual understanding using the respective assessment sections or performing additional activities on their own (Figures 2F and 3E). On the home page of the training resource, we provide links to videos showcasing the teaching of a module.

For online teaching, we found that it is hard for learners with only one screen to click‐along or type‐along. In this case it is best to showcase the activity and ask the learners to either repeat the activity or perform the subsequent activity. In this situation it is important to leave enough time for feedback at the end of each activity.

For more detailed guidance, please refer to our TEACHING_TIPS.md in the GitHub repo.^13^

## 6 HOW TO CONTRIBUTE

The implementation of the training resource relies on modified code from *The Carpentries* for building the training module websites (https://neubias.github.io/training‐resources/index.html). All raw material and source code is hosted on GitHub https://github.com/NEUBIAS/training‐resources/ ^13^ enabling collaborative development by a wide community. Using Jekyll,^20^ all modules are converted to HTML and can be accessed by any common web browser (Figures 2 and 3). The material is CC‐BY licensed such that it can be re‐used freely with acknowledgement of the original source. GitHub issues are used for discussions about adding new material or improvements of the current content. For example, https://github.com/NEUBIAS/training‐resources/issues/256 features an interesting discussion on how to explain the ‘watershed’ algorithm. Pull requests streamline the review and integration of new content. Contributors can not only develop new or enhance existing modules, but also provide valuable resources like example image data for activities and ideas for additional activities. Details about how to contribute are documented in CONTRIBUTING.md in the GitHub repo.^13^

## 7 DISCUSSION AND OUTLOOK

Currently, existing online material mainly focuses on self‐study, lacks modularity, does not allow to flexible reorganise topics, and is typically hard to further develop by the community.^4^, ^5^, ^6^, ^7^, ^8^, ^16^

Our training resource aims to complement existing material by supporting instructors in delivering live courses (online and/or in person). The courses address researchers at the level of master or above that want to solve bioimage analysis problems. Live courses allow for networking, interactivity with the instructors and other learners, as well as project work. Our resource has been used by numerous trainers to educate hundreds of students over several years. Its modular structure offers several practical benefits. Beside courses, we used the resource to provide supporting information during project discussions between bioimage analysts and their collaborators.

As individual units, modules can be easily added or updated without disrupting the overall structure, making contribution by the community easy. While careful consideration should be given to how new material aligns with existing content, minor overlaps can be accommodated. This modularity also simplifies course preparation for multiple trainers, as each can focus solely on their assigned modules. Given the streamlined nature of each module, the preparation effort for a trainer is minimal. This is a significant advantage, as it facilitates the onboarding of new trainers and contributes to expanding the global bioimage analysis teaching workforce.

The activity in each module typically illustrates the module concept using microscopy images of biological samples, making the resource interesting for scientist using microscopy in their research. The focus on software agnostic modules makes it possible to use the same training material to design various courses, targeting different audiences, and using different software. For instance, both researchers working with point scanning fluorescence microscopy or with colour camera based histological imaging data will need to know the content of the ‘digital image’ module and also more advanced modules such as ‘distance transform’ or ‘object shape measurements’ are likely relevant to both audiences. However, while the concept of ‘colour space’ is critical for histological imaging data it is irrelevant for fluorescence data. Thus, when creating a course for researchers working with histological data, it may only be necessary to add a new module about ‘colour space’ and some new activities about RGB images in the existing ‘datatypes’ module, while many other parts of the course could be covered with existing modules.

Our approach also has disadvantages when compared to others. Although many modules provide references and links for further reading, our material emphasises examples and practical application to microscopy images of biological samples over exhaustive completeness or deep mathematical details. As a module covers the theoretical foundations of a topic only very concisely, learners will need to run some of the activities to gain a deeper understanding. This makes self‐study harder, as learners might miss crucial context and explicitly need a software installation.

A further challenge of our modular approach is deciding on the granularity of teaching content. This means carefully assessing what to include in one module versus splitting into several. This decision impacts how appealing the material is to different trainers and audiences. While our framework allows modules to co‐exist, even with redundant content, we've avoided duplication so far. However, this might become an option if it makes the resource more attractive to a wider audience.

We are aware that the topics covered by our training material only address a minor fraction of the bioimage analysis field. We try to teach each concept using a generic terminology, however, the choice of example images has a bias towards light microscopy, reflecting the fact that many of the authors work within a light microscopy facility. Nevertheless, we believe that we do cover the essentials. The topics taught by other resources mainly cover ‘data handling’ skills (e.g. opening, saving, inspecting, dealing with different data structures and formats), and skill on how to extract quantitative data from the images (e.g. filtering, segmentation, object shape and intensity measurements).^5^, ^7^, ^8^, ^16^ Thus, there seems to be an agreement among the community as what is considered an essential bioimage analysis skill. Indeed, a fraction of our modules is already sufficient to cover these essential topics.

We do not want to limit our resource to basic topics and are continuously working on new (advanced) material and courses. In particular, image acquisition and degradation is relevant to assess the reliability of measurements. For instance, although you can measure object shapes, one should be aware of the resolution limit and which metrics are still reliable. In many modules and their corresponding activities, we discuss images that have potential issues, the origin of these issues, and how to compensate those (e.g. data types and image saturation, low SNR in intensity quantification, local background/uneven illumination). We believe that this provides microscopists guidance on how to beast record images for quantification. In addition, we provide several modules, consolidated in a course, on light microscopy image formation (e.g. point spread function, resolution) and degradation (e.g. spherical, stitching artefacts), and how those may affect image analysis.

One important aspect of bioimage analysis is the validation of the results and currently we have two modules on this topic: a module illustrating batch processing of the results for visual inspection and a module introducing quantitative metrics for comparison with ground truth data (e.g. intersection over union). This is appropriate for the current status of the teaching material, where we only use simple algorithms for segmentation. In the context of machine learning, one would require additional metrics (e.g. a confusion matrix) to assess the errors. We are developing modules to introduce students to machine learning based methods for segmentation, image classification and image restoration. Within this effort we plan to develop modules on data validation. We also miss modules to quantify colocalisation and co‐occurrence, a topic dear to molecular biologists, and we also lack modules on tracking. Due to the ever‐increasing amount and complexity of image data we are also working on adding more modules and eventually a course related to image data management. Finally, the material could address different imaging modalities. Currently, we have only few examples with data from electron microscopy and no modules using data from magnetic resonance imaging (MRI) or computer tomography (CT). Thus, much training material could and should be added. In this context we would like to emphasise again that the modular open‐access nature of our resources facilitates and invites addition of missing material by the growing community of bioimage analysts.

Our resource mainly concentrates on ImageJ and Python implementations for the activities. However, there are many other open‐source image analysis software (e.g. Icy, Cellprofiler, Knime) and commercial software (e.g. Matlab, Arivis, Imaris). It would be nice to add additional software implementations and this is readily possible without disruption the existing material.

An important aspect of the resources is suitable example image data, which is currently hosted in the same GitHub repository as the training material. While this is convenient, it does not allow to host large image data. We are therefore considering for the future to host training image data on an image repository, for example, the BioImage Archive.^21^

In conclusion, the presented resource with its modularity allows to teach an evolving field such as bioimage analysis. The resource has been used to teach students at different levels by multiple trainers in various courses and we are positive that the training content and also the number of trainers will keep increasing in the coming years. We therefore foresee our open‐access resource to make a valuable contribution to satisfying the global need for bioimage analysis courses.

## Supporting information



Supporting information

Supporting information

Supporting information

Supporting information

Supporting information

Supporting information

Supporting information

Supporting information

Supporting information

## Data Availability

The data that support the findings of this study are openly available in Bioimage Analysis Training Resources at https://neubias.github.io/training‐resources/, reference number https://zenodo.org/records/14710820.
