# Supplementary material for: Modular training resources for bioimage analysis
Source: J Microsc. 2026 Jun 18;304(1):3–14. doi: 10.1111/jmi.70133 (PMC13644699; doi:10.1111/jmi.70133)
Supplement: Supplementary file 1 [file JMI-304-3-s002.pdf]

Event name	Host	Starting date	Participants	Trainers	Location	Main organiser(s)
Basics of Bioimage Analysis	EMBL & MPI Multidisciplinary Sciences, Germany	January 25, 2021	40	2	Virtual	Christian Tischer, Antonio Politi
Basics of BioImage Analysis	EMBL, MPI Multidisciplinary Sciences	June 7, 2021	40	2	Virtual	Christian Tischer, Antonio Politi
Basics of BioImage Analysis	EMBL, MPI Multidisciplinary Sciences	March 8, 2022	50	2	Virtual	Christian Tischer, Antonio Politi
Basics of BioImage Analysis Scripting	EMBL, MPI Multidisciplinary Sciences	March 21, 2022	50	2	EMBL Heidelberg	Christian Tischer, Antonio Politi
Basics of bioimage analysis using Fiji and scripting	MPI Medical research, Heidelberg	December 7, 2022	20	1	MPI-MR Heidelberg	Antonio Politi
EMBO Practical Course: C. Elegans: from genome editing to imaging	EMBO	July 17, 2022	20	2	EMBL Heidelberg	Henrik Bringmann, Simone Köhler, Georgia Rapti, Pavak Shah
Basics of image data inspection	EMBL Predoc course	October 24, 2022	18	2	EMBL Heidelberg	Simone Koehler
Basics in Bioimage Analysis, IMPRS, MolBio Master Course	MPI-NAT, Goettingen	October 18, 2022	12	1	MPI-NAT, Göttingen	Antonio Politi
Basics in Bioimage Analysis, GGNB Master course	MPI-NAT, Goettingen	October 27, 2022	12	1	MPI-NAT, Göttingen	Antonio Politi
Basics of image data handling & inspection	ALMF/EMBL	January 19, 2022	16	2	EMBL Heidelberg	ALMF
Basics of image data handling & inspection	ALMF/EMBL	February 7, 2022	16	2	EMBL Heidelberg	ALMF
In situ CLEM at Room Temp. and in Cryo	EMBL	February 12, 2023	20	1	EMBL Heidelberg	Martin Schorb
Defragmentation TS2	NEUBIAS	May 8, 2023	35	1	Porto, Portugal	Paula Sampaio, Rocco D'Antuono
Image analysis using skimage and napari	EMBL, MPI Multidisciplinary Sciences, FMI Basel	June 6, 2023	50	5	Virtual and in person at participating institutions	Christian Tischer, Antonio Politi, Tim-Oliver Buchholz
ABiS-GBI 2023 course - Image data: image analysis, data management and reuse	GBI	July 3, 2023	15	3	Okazaki and Kobe, Japan (NIBB and RIKEN BDR)	Gleb Grebnev
Basics of image data handling & inspection	ALMF/EMBL	January 30, 2023	32	1	EMBL Heidelberg	ALMF
Basics in Bioimage Analysis, IMPRS, MolBio Master Course	MPI-NAT, Goettingen	November 7, 2023	12	1	MPI-NAT, Göttingen	Antonio Politi
Basics in Bioimage Analysis, GGNB Master course	MPI-NAT, Goettingen	November 23, 2023	12	1	MPI-NAT, Göttingen	Antonio Politi
Basics of bioimage analysis in python	EMBL	April 29, 2024	20	4	EMBL Heidelberg	Christian Tischer
GBI-LNMA 2024 course: Image analysis and data reuse through image repositories	GBI	October 6, 2024	25	1	Centro Internacional de Ciencias AC, UNAM	GBI (Gleb Grebnev)
Basics of bioimage analysis in python	IDIP (CIID)	January 7, 2024	17	6	CIID Heidelberg, Germany	Christian Tischer, Severina Klaus
Python based image analysis using skimage and napari (RTG CYTAG, PhD Course)	MPI-NAT, Goettingen	June 12, 2024	12	1	MPI-NAT, Göttingen	Antonio Politi
Batch image analysis in python	EMBL	June 8, 2024	12	4	EMBL Heidelberg & Zoom	Christian Tischer
Basics in Bioimage Analysis, IMPRS, MolBio Master Course	MPI-NAT, Goettingen	November 5, 2024	12	2	MPI-NAT, Göttingen	Antonio Politi, Monica Gobran
Basics in Bioimage Analysis, GGNB Master course	MPI-NAT, Goettingen	November 21, 2024	4	2	MPI-NAT, Göttingen	Antonio Politi, Peter Lenart

Supplemental Table 1: List of courses 2021-2024 that use the training resource
