# Supplementary material for: Modular training resources for bioimage analysis
Source: J Microsc. 2026 Jun 18;304(1):3–14. doi: 10.1111/jmi.70133 (PMC13644699; doi:10.1111/jmi.70133)
Supplement: Supplementary file 2 [file JMI-304-3-s006.pdf]

Course	Synopsis	Modules	Teaching time (h)	ECTS credits
Bioimage Inspection	Learn to inspect your images and visualize your data.	Digital image basics<br>Data types<br>Display settings<br>Spatial calibration<br>Multichannel images<br>Projections<br>Volume slicing<br>Quantitative image inspection and presentation	8	0.4
Bioimage Analysis	Learn essential concepts of image analysis, such as segmentation, intensity measurements, and filtering.	Image analysis course<br>Segmentation overview<br>Thresholding<br>Automatic thresholding<br>Connected component labeling<br>Object shape measurements<br>Nuclei segmentation and shape measurement<br>Object intensity measurements<br>Neighborhood filters<br>Statistical (rank) filters<br>Local background correction<br>Convolutional filters<br>Morphological filters	9	0.6
Advanced Topics in Bioimage Analysis	Further improve your skills in image analysis.	Object filtering<br>Distance transform<br>Watershed<br>Nuclei and cells segmentation<br>Skeletonization<br>Correlative image rendering	4.5	0.3
Image Data Formats	Learn how to open and handle different image data formats.	mage data formats course overview<br>Image data formats<br>Big image data formats<br>OME-TIFF<br>OME-Zarr	3	0.2
Bioimage Batch Analysis	Learn how to automatically process image analysis tasks on multiple images.	Batch analysis course overview<br>Loops<br>Strings and paths<br>Functions<br>Batch processing<br>Batch exploration of segmentation results	3.75	0.25
Bioimage Batch Analysis Essentials	Crash course to learn the essentials of how to devise a basic image analysis workflow and how automatically apply it on multiple images.	Nuclei segmentation and shape measurement<br>Batch processing	2	0.13
Cloud Based Bioimage Analysis	Learn about executing bioimage analysis on remote compute	Cloud based bioimage analysis course overview	1.5	0.1

	resources.	Cloud based interactive analysis<br>Cloud based batch analysis		
ImageJ Macro	Learn how to generate and modify scripts for image analysis workflows using the ImageJ macro language.	Running a script<br>Recording a script<br>Variables<br>Loops<br>Strings and paths<br>Handling input parameters<br>Output saving<br>Batch processing	6	0.4
Image formation and imaging artefacts	Learn some essential concepts on what determines quality and resolution of images, how this affects quantifications, and how to mitigate imaging artefacts	Point spread function<br>Image sampling<br>Resolution<br>Optimal sampling<br>Optical sectioning<br>Imaging thick biological samples<br>Spherical aberrations<br>Sample preparation artefacts<br>Illumination and shading artefacts<br>Flat-field correction<br>Stitching of tiled images	9	0.6

Supplemental Table 2: List of consolidated courses
