# Supplementary material for: Modular training resources for bioimage analysis
Source: J Microsc. 2026 Jun 18;304(1):3–14. doi: 10.1111/jmi.70133 (PMC13644699; doi:10.1111/jmi.70133)
Supplement: Supplementary file 3 [file JMI-304-3-s007.pdf]

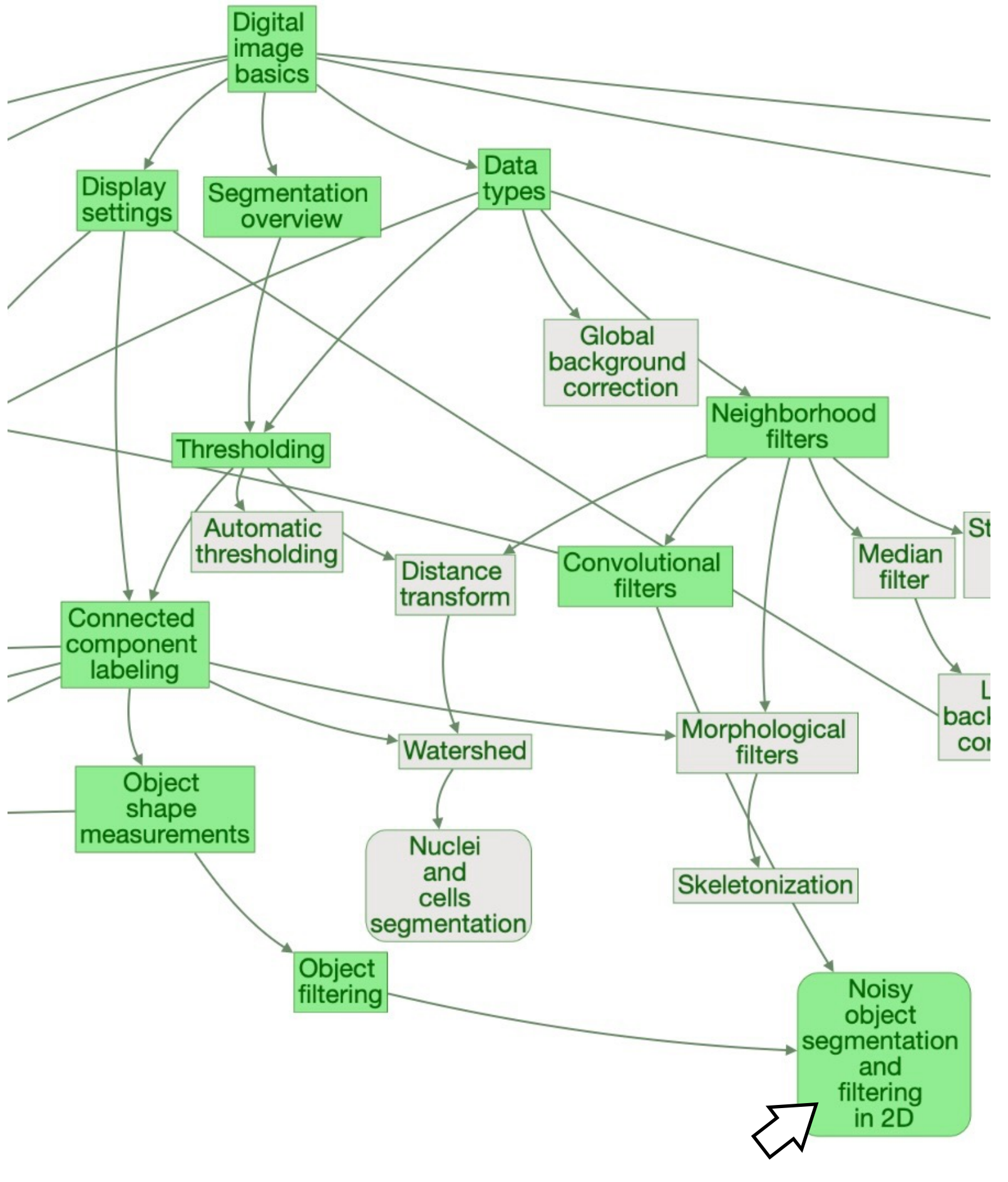
Digital image basics
Display settings
Segmentation overview
Data types
Global background correction
Neighborhood filters
Thresholding
Automatic thresholding
Distance transform
Convolutional filters
Median filter
Connected component labeling
Watershed
Morphological filters
Object shape measurements
Nuclei and cells segmentation
Skeletonization
Object filtering
Noisy object segmentation and filtering in 2D

Figure S1
