# Supplementary material for: Modular training resources for bioimage analysis
Source: J Microsc. 2026 Jun 18;304(1):3–14. doi: 10.1111/jmi.70133 (PMC13644699; doi:10.1111/jmi.70133)
Supplement: Supplementary file 5 [file JMI-304-3-s005.docx]

## Video transcript to the manuscript **Modular training resources for bioimage analysis**

Teaching of the module [Digital Image Basics](https://neubias.github.io/training-resources/pixels/index.html), introduction

Speaker: Christian Tischer, EMBL Heidelberg, Germany

### Transcript

Hello, everybody,

My name is Christian Tischer. I'm the head of the Bioimage Analysis Support Team at EMBL. I will be presenting a training module for Bioimage Analysis about digital image basics. For software installation instructions and further information, please read the description of the video.

So the learning objectives are to understand that the digital image is represented as an n-dimensional array, to be able to practice practically visualize an image and also practically learn how to quantitatively inspect the elements of an image, which are called pixels in 2D for picture elements and voxels in 3D for voxel elements.

So why is this important? Well, technically, one can say digital images are a very important subset of a more general mathematical definition of an image. And it is a very important one because, to our best knowledge, the vast majority of available algorithms and visualization tools operate on digital images and also scientific microscopes. As far as we know, always output digital images. Thus, in summary, if you are interested in microscopy-based science, it is really crucial to understand the basic properties of digital images.

So here is a little concept map. So a digital image, as mentioned, is just an n-dimensional array which has elements and it has a data type. This is, however, something we will cover in another module in more detail. And it also has a certain size. So here is a practical example of a very simple image of a fluorescent nucleus. So on the right, you maybe see the familiar representation as a grayscale image where bright pixels correspond to high values, which then correspond to more fluorophores, for example, in the actual sample. We can, for example, try to find here the brightest pixel, which is probably this one, and then see here on the left this corresponds to an actual value of 68, which again is somehow proportional to how many fluorophores there were.

In addition to the actual values of the image, it's also very important to observe that there are pixel indices. So these are the green values here, and this is important to precisely refer to one specific pixel. So for example, here the brightest pixel in the image with a value of 68 lives at the indices of 9 and 5. What we can also immediately see here is the size of the image. So it's an image that has 21 by 21 pixels. This doesn't have to be square. In fact, many microscope cameras also output non-square images, so it could be 1000 by 2000 pixels or anything else. If you have three dimensions, we would simply add another dimension to this array. Unfortunately, one very typical source of confusion is to which pixel do these indices really refer, and there are different conventions here. I'm starting with counting in the top left corner of the image, which I think is mostly the case, but maybe some softwares might start in the lower left corner, although I think upper left is really the majority. Last agreement is on the topic whether you start counting with 0 or 1. So the top left pixel could be at coordinates 0, 0 or 1, 1, depending on the software that you're using. So you have to be very careful, and then correspondingly everything here will be shifted by one pixel. And then again, unfortunately, it may differ whether you first say the 9 and then the 5 or you first say the 5 and then the 9. So you have to be really careful when you want to refer to one specific pixel. You have to basically say which convention you're using or maybe which software you used to make the statement. Okay, I think that's it for the introduction, and then we will continue with really learning using a specific software how to open such an image and expect its values.

### Transcript

So hi. We will now do an activity of the digital image basics module. We will do the first activity, which is called inspect a two-dimensional image. We will do this using Python, and more specifically, the skimage library and the Napari viewer.

So here is the code that you can also execute yourself. I will now execute it step by step and explain everything. For this, I will remove this window here and then in my terminal window, where I already have a Conda environment activated with all the necessary dependencies, I will start the Jupyter notebook. In the notebook, we need a Python.

All right, so the first thing we want to do is actually open an image and for this, we will use a library that's called open.ij.tiff. We will use the similarly named function, which can open images that have been saved, TIFF images that have been saved with ImageJ. Then we will use a web URL that is very long. And that's why I will copy it and put it into the image URL variable. To load this, we use the open.ij.tiff function, which accepts the image URL, which we will then put into a variable, which we just call image. This function also outputs some other things that we will only need in later training modules. To ignore them, you can use this syntax in Python, this will basically swallow everything that is outputted after the image.

Well, let's see. OK, that seems to have worked. The open.ij.tiff function is telling us that this image is not calibrated, something we will learn more about in other modules. For now, we can just live with this. So now what is actually this image? To see that, we can say type. This gives us the actual type of this image variable. We see it's a NumPy array, which is maybe not a surprise. This is what's typically used in Python to represent arrays.

We can also get a glimpse on the values in this image. For this, we can simply actually print the image itself. We see some of the pixel values and we also see the data type here, which is unsigned integer bit. Again, something we will learn more about in other modules that are specifically concerned about data types. Another interesting thing is to check how large the image is. For this, we can look at the shape that this is called in Python, NumPy. OK, relatively small. It's 50 by 59 pixels.

Now, we looked so much at this number, we want to actually see it, right? And as promised, let's use Napari. So we need to import it from napari.viewer. Import viewer and then we need to instantiate a viewer. We want also a handle on it, such that we can interact with it and this is how this works. This will instantiate one viewer and this variable here will be a handle, this napari.viewer. Now, let's add the image to it. So this is very intuitive. So you say, viewer.addImage. I'm using tab here, auto-completion. Always a good idea. Let's just add the image. All right, beautiful. We see this is a small image, which has two nuclei, one that is a little bit dimmer and one that is a little bit brighter.

Now, if we hover here over the image, we can actually see the pixel values. Here is a rather bright one, 123, 129. And then in the background, we have values around 50. And the two numbers before are the indices of these pixels. So here it's important to check what the axis order is. So if I go up and down, the first index changes. And if I go left and right, the second one changes. So this is the first axis. This is the second axis. This is very important, because, for example, in ImageJ, unfortunately, it's the other way around. So you always have to check this.

So how to extract now single pixel values from the array? So for example, we could be interested in a pixel up here, maybe 68. OK, this is actually done like this. So square brackets, and then you give the indices that you are interested in. And indeed, 63 is correct. This is also what we saw in Napari. As a control, let's maybe take a really bright one. So I actually always have to click with the left mouse button. 3142. Let me take a note here. 3142, and the value should be 95. OK, let's try. Image 3142. 31. 31. 31. Yeah, 123. Sorry, 95 was wrong. I made the wrong note. OK, perfect. Everything's good. Always be skeptical. Another useful thing sometimes is to extract one line of an image. Maybe here we can use that goes across both nuclei. So this would be good here, around a value of 20 in the first dimension. So we can do this by this syntax. Again, square brackets, and then we want row 20, and then all values in this row. So we can execute this. And then we get these values. And then you can see we first have a bunch of rather dim values. So this corresponds to this large background region. Then a few brighter ones. This is the nucleus here. And again, some dim ones, which again is this stretch. And then the second nucleus comes here. And again, some background. So it's always critical to check, do these values that you get in the code correspond really to what you see in the image? Because often you're just making some mistake, wrong dimension order, something. So always check.

What can be also useful is to extract crop here, maybe one nucleus. So let's check. For example, this nucleus starts at row 8 and stops at row 30. I would try to make a note again, 8 to 30. And the columns are 11 to 25. So to extract this, we can type image square brackets, rows 8 to 30, and then columns 11 to 30. 2, 5. So here we extracted a subset of this array. And you can actually appreciate that here in the middle, we have the brightest values surrounded by dimmer background values around 50. OK.

Another very useful thing is to ask what are the brightest and dimmest values in such an image. So this you can do in the following way. You can print. So we type a function called min. Since it's a function, you need brackets. And also, it's a corresponding max function. So we can execute this, and we see here what the dimmest and the brightest value is in the image.

Another useful thing is how to get an image actually from Napari in the code. And you can do this by referring to the, for example, by the name that you typed here, which, in fact, one can also change. You can, for example, call this Napari image. And to now fetch this back into your code, you have to type the following. Viewer. So this is our instance. And then layers. This corresponds to this here. So at the moment, we have only one layer, but there could be several. Now we have to say which layer. So this is an array. So we index it with the name, Napari image. And of the layer, we want the data. This is important. And we want to store all of this in a variable, which we maybe also call Napari image.

So all right. And then now it's important to check maybe that this is really the same data that we added. And for this, we can also use another very useful function that we can introduce here from NumPy. So we actually have to import NumPy. And typically, one uses the abbreviation np. And we can then use NumPy.array equal and ask, is our original image the same as the image that we added into Napari? And lo and behold, they are the same. And actually, they don't only have the same values, but they are really the same arrays, it turns out. So to test this, you can actually ask in Python, Napari image, is image? And that tells you they are really not having only the same values, but are the same object. That can be very important, because it means if you change one of them, both of them will change, because they point to the same memory in your computer. Might be a bit advanced, but we thought this is a good moment to mention this.

All right, and then finally, what people typically do, and what people also, ourselves, right, bioimage analysts, it's to get an overview of all the values in an image. And for this, we want to plot a histogram. And actually, since this is a bunch of code, I just will copy it, because it's hard to remember anyway. So typically, you will probably copy it from somewhere. So actually, in NumPy, we already import it, so we can remove this. So here, we need a new library for plotting, which is from matplotlib, which is a very classic plotting library. And then we say, please make a histogram of all the values in the image. And to do this, we actually have to make it a one-dimensional image. This is what the flatten is doing. And then we also have to say from where to where. And we do this from the minimum to the maximum of the image. Plus 1 is a typical computer thing, so we need this that we have actually a bin also for the maximum value. That's why it's one more. And then we just show it. And this actually gives you here, in the notebook, a histogram of the image. So you see, most of the values are around 50, which again, we can check here is the background. And then actually, the bright values here, which are the nuclei, are not so many. So it's very interesting. Our brain, I think, often overperceives the bright objects. You see they are actually rather the minority. So this will be very important later if you, for example, want to segment images, because then you need to often threshold an image and say everything that's brighter than something is an object. And for this, you always look at these histograms. That's it for this activity. Thank you very much for listening. And I will stop here.
