# Supplementary material for: Modular training resources for bioimage analysis
Source: J Microsc. 2026 Jun 18;304(1):3–14. doi: 10.1111/jmi.70133 (PMC13644699; doi:10.1111/jmi.70133)
Supplement: Supplementary file 6 [file JMI-304-3-s009.docx]

## Video transcript to the manuscript **Modular training resources for bioimage analysis**

How to use the Bioimage analysis training resource

Speaker: Antonio Politi, MPI-NAT Göttingen, Germany

Hello, my name is Antonio Politi.

I am a Bioimage Analyst working for the Max Planck Institute for Multidisciplinary Sciences in Göttingen, Germany. In this video, I will present to you the Bioimage Analysis training resources we developed with several other experts in the field. This video is meant for instructors that want to use this training resource for their own courses. You can find further details and links in the description.

You can reach the training material using a browser by typing in the address of the training material. The elementary unit of the training resource is a module. Over the years, we developed over 50 different modules that explain essential and important concepts in Bioimage Analysis. Typically, modules are taught within a course and on the landing page you can find several course suggestions that give you the number of modules we use, the approximate time needed, and how many credit points.

For instance, if you click on Basic Bioimage Analysis, you get a set of modules that you can teach. This should be enough for teaching Basic Bioimage Analysis. If you want to develop your own courses, you can of course browse through the long list, but you can also use the module dependency network, which is also on the landing page, where you can find all the modules as nodes. If you click on a module, for instance, if you say you want to do a segmentation of noisy objects in 2D, then you can click on the module and this will highlight all the modules that you need to be able to perform this task. You can also control or shift-click on one of these objects and then, for instance, shift-click will bring you to the module and have a look at it.

Let us look into more detail how a module is structured. Click, for instance, on one of the courses and one of the modules. You can see that we land on the module page. The module typically is dedicated to teach one or very few concepts. For instance, in this module, it's all about thresholding. Modules are subdivided in different sections. You will have a prerequisite section that defines which modules are needed before the current concept and for the current module. A learning objective defines what learners should have understood at the end of the module. Motivation gives the reasons why it is important to learn the current concept. A concept map is meant to show in a very concise way what are the important parts that learners should understand to understand, for instance, thresholding. Finally, a figure helps the instructor to illustrate the concept in a graphical way. Typically, when teaching, the instructor shows the figures and sometimes the concept map. By using the information included in the module and the motivation and learning objectives, it tries to explain the concept in this module in a very generic way so that it's understandable. This is more like a lecturing part of the teaching module.

This generic explanation is then followed by one or several activities that are executed live by the instructor. You can find activities at the end of this first section that may contain also further explanation to better understand the concept. There can be one or several activities. An activity has always an image connected that you can either drag and drop on an image analysis software or download by clicking on it. An activity has a description of what the instructor is supposed to do or learners are supposed to do for this specific activity. As you can see, the instructions are rather generic and this is on purpose because they should be agnostic for a specific software implementation. If you click on Show Activity, then you will get a list of implementations for this activity. For instance, ImageJ GUI, this is a list of things you need to click and some explanation for this activity. The instructor in this case will not show the website but will share with the students a screen that contains ImageJ where it then really executes this task in front of the students.

Typically, you want to execute this task in a slow pace so that students can click along. Slow enough that you can click and repeat with the instructor exactly the same task. We also have often code implementations. For instance, Python is a very useful image analysis software or language that's used for image analysis. In this implementation, we use skimage and Napari. Here again, the instructor will use, for instance, JupyterLab to then showcase this activity. We found it is not very useful to just copy everything and then execute the activity in one go, but it's rather better to copy either small parts or even type small parts explaining what the comments do, executing the commands and explaining the results. This is the right pace for students to follow the instructor.

These activities can also be executed independently by the learners and in this case, of course, they can refer to this text and then really type and/or copy paste themselves this code to do it. At the end of the activity session, you often find an assessment or formative assessment section and this is meant for learners to further reinforce what they have learned. This can be done in the class or they do it independently and learn about this. Finally, there are some links for follow-up material that should help you know what to learn next.
